# Generative Artificial Intelligence Literacy Scale for Nurses: Development and Psychometric Evaluation

**DOI:** 10.2196/95547

**Published:** 2026-07-06

**Authors:** Kuan-Lin Chu, Ching-Ling Wang, Che-Ming Chang, Jyh-Chong Liang, Lu-Yen Anny Chen, Chieh-Yu Liu, Cheng-Pei Lin

**Affiliations:** 1 Department of Nursing Taipei Veterans General Hospital Taipei Taiwan; 2 Institute of Clinical Nursing, College of Nursing National Yang Ming Chiao Tung University Taipei Taiwan; 3 Department of Nursing Taipei Hospital, Ministry of Health and Welfare Taipei Taiwan; 4 Department of Nursing Mackay Memorial Hospital Taipei Taiwan; 5 Program of Learning Sciences and Institute for Research Excellence in Learning Sciences National Taiwan Normal University Taipei Taiwan; 6 Institute of Community Health Care, College of Nursing National Yang Ming Chiao Tung University Taipei Taiwan; 7 Cicely Saunders Institute of Palliative Care, Policy & Rehabilitation King’s College London London United Kingdom

**Keywords:** artificial intelligence, generative artificial intelligence, literacy, nurses, psychometrics, surveys and questionnaires

## Abstract

**Background:**

Generative artificial intelligence (GenAI) can automate time-intensive tasks and support clinical decision-making in care settings. Nurses require appropriate competencies to ensure that integration of GenAI strengthens care quality and patient safety. However, validated literacy assessment tools remain limited. In particular, instruments tailored to nurses’ role-specific GenAI competencies, including hallucination detection, risk identification, and ethical accountability, are lacking. These gaps highlight the need for a nurse-specific GenAI literacy scale.

**Objective:**

This study aimed to develop and psychometrically validate the Generative Artificial Intelligence Literacy Scale for Nurses (GenAILS).

**Methods:**

We conducted a two-phase, cross-sectional online survey of registered nurses nationwide in Taiwan between June 2025 and October 2025. Phase 1 involved conceptualization and item generation based on a literature review, followed by content appraisal through expert discussion with 6 external reviewers. A 50-item pool was generated. Subsequently, 5 external reviewers evaluated content validity. Items with a content validity index of <0.78 or flagged for revision were revised or deleted. Phase 2 evaluated psychometric properties (item analysis, internal consistency, split-half reliability, and criterion-related validity) and construct validity via exploratory factor analysis (factor loading ≥0.60), followed by confirmatory factor analysis (CFA). The total sample was randomly split into 2 independent subsamples for exploratory factor analysis and CFA.

**Results:**

In phase 1, the initial 50 items underwent expert content validation and were revised to 46 items (scale content validity index based on the average method=0.92). In phase 2, 1313 questionnaires were collected, of which 191 invalid responses were excluded; 1122 valid responses were analyzed. Participants had a mean age of 34.66 (SD 7.8) years. Extreme-group comparison revealed statistically significant differences for each item (*P*<.001). The final scale comprised 24 items across six dimensions: responsible use, updated competencies, risk identification, fundamental knowledge, critical evaluation, and ethics and law. The cumulative variance explained was 53.1%. The first-order CFA demonstrated excellent model fit: root-mean-square error of approximation=0.035, standardized root-mean-square residual=0.032, comparative fit index=0.99, goodness-of-fit index=0.94, adjusted goodness-of-fit index=0.93, nonnormed fit index=0.99, and parsimony normed fit index=0.84. The second-order CFA demonstrated excellent model fit: root-mean-square error of approximation=0.039, standardized root-mean-square residual=0.040, comparative fit index=0.99, goodness-of-fit index=0.94, adjusted goodness-of-fit index=0.92, nonnormed fit index=0.99, and parsimony normed fit index=0.87. All heterotrait–monotrait ratio values were below 0.85, supporting discriminant validity. The scale was moderately correlated with the Short Form Meta-AI Literacy Scale (*r*=0.57; *P*<.001). Reliability was excellent (Cronbach α=0.92; McDonald ω=0.92; split-half reliability=0.81).

**Conclusions:**

The GenAILS is a concise, nurse-specific self-report instrument with good psychometric properties across 6 clinically relevant domains. It supports needs assessment, targeted training, and intervention evaluation to promote the safe and ethical use of GenAI in nursing.

## Introduction

The rapid evolution of artificial intelligence (AI) has established AI literacy as an indispensable core competency in modern society and professional education. The rapid advancement of generative artificial intelligence (GenAI) has fundamentally transformed how knowledge is acquired, information is processed, and decisions are made [1]. GenAI has introduced transformative changes in health care. Earlier systems were primarily limited to classification or early-warning functions; however, GenAI can draft nursing care records, automatically generate handover summaries, and streamline complex administrative paperwork to support nurses proactively. These capabilities substantially reduce cognitive workload and time burden. In patient care, GenAI-powered conversational agents can deliver empathic psychological support and provide timely, tailored health education, thereby improving nurse-patient communication. This technological advancement simplifies nursing tasks, alleviates workload, and facilitates greater integration across care processes, enabling nurses to devote more attention to direct patient care. GenAI may also help mitigate workforce shortages and occupational burnout, ultimately improving overall health care quality [2-4]. Consequently, incorporating GenAI into nursing education is crucial to respond to the rapid evolution of clinical nursing practice, which integrates critical thinking and professional values [5].

To ensure safe and appropriate application, distinguishing the operational nature of GenAI from discriminative AI is essential. Unlike discriminative AI, which primarily focuses on classification, prediction, and recognition, GenAI is characterized by its capacity to generate novel content. It can autonomously produce multimodal outputs (eg, text, images, audio, and code) that resemble human creativity [6,7].

GenAI can be conceptualized using a three-layer structure: (1) the model, comprising general-purpose foundation and customized models that generate content; (2) Connection, enabling deployment and system interoperability via fully integrated solutions or application programming interface-based integration; and (3) Application, supporting diverse end-to-end uses in clinical and educational settings [7]. However, this structure introduces unique risks. GenAI’s generative capability may lead to hallucinations, producing incorrect or misleading outputs presented plausibly and convincingly [8]. Users must possess advanced identification and coping capabilities to navigate the complex ethical and legal landscape, including copyright, data originality, and algorithmic bias [9,10]. Without a thorough understanding of its mechanisms, nurses may accept erroneous outputs, compromising patient safety and jeopardizing care plans [11,12]. Health care providers are pivotal in translating GenAI into safe clinical practice, highlighting the need for enhanced GenAI literacy to identify risks, apply safeguards, and ensure accountable use [8,13]. Therefore, the definition of literacy must evolve. Although literacy is traditionally defined as the integrated ability to understand, use, and apply specific information [14], GenAI literacy refers to the competencies required to understand its fundamental principles, functions, and capabilities, adapt to its evolving nature, and apply the technology responsibly and ethically to produce consistent, creative, and standards-aligned outputs, such as newly generated text, images, and audiovisual content [1]. Importantly, GenAI literacy extends beyond general AI literacy by emphasizing critical appraisal of AI-generated outputs, including validating accuracy and clinical appropriateness, detecting bias and hallucinations, and managing misuse-related risks—competencies essential for clinical nurses [1,8,13].

GenAI literacy is distinct because it integrates foundational understanding, responsible use, and continuous learning. It requires users to recognize GenAI as a content-generating technology rather than a traditional search tool and to move beyond basic operations by addressing ethical issues, critically evaluating output accuracy, and managing privacy and security risks [1]. With increasing GenAI use among patients seeking health information and health care professionals for communication, education, and decision-support, its generative nature introduces risks that general AI literacy frameworks do not fully capture. Therefore, GenAI demands tool-specific competencies, particularly critical evaluation. Without a specialized GenAI literacy framework, nurses may remain underprepared for AI-integrated health care [1,8]. Existing AI literacy frameworks are often too generic, originate from non-nursing fields, or fail to address GenAI’s specific usage characteristics and risk profiles, such as hallucination in clinical decision support and data privacy concerns related to patient records [15-17].

Despite the urgency of integrating AI into nursing curricula, training methods remain inadequate, and significant gaps exist in the empirical assessment of these skills. Nurse educators must integrate AI-related content into existing curricula to prepare learners for a digitized clinical landscape [18]. However, accurate assessment of learners’ current AI literacy is a prerequisite for effective curriculum design. Medical professional literacy has shifted from technical development to everyday interaction and application [19]. As GenAI applications become increasingly prevalent in clinical nursing, systematic integration of AI literacy into nursing education is an urgent priority. Literature indicates that up to 84% of health care professionals are optimistic about GenAI’s potential to improve health care [20]. Nursing education must therefore evolve by integrating AI content into core curricula and enhancing GenAI literacy among educators and students [16,20,21]. Because erroneous or unverifiable GenAI outputs can propagate into documentation and decision support, nurses require verification competencies to mitigate patient-safety risks. The Generative Artificial Intelligence Literacy Scale for Nurses (GenAILS) can support competency benchmarking to inform education, governance, and safe implementation pathways for generative AI in clinical nursing.

Although GenAI has immense potential to reduce administrative burdens, support nursing workflows, and enhance evidence-based practice [5,22], its safe and ethical use depends on human judgment. Most AI literacy scales focus on general learners and do not adequately address the decision-making needs, patient safety considerations, and ethical norms unique to the nursing clinical context. A nurse-specific scale is needed because nurses’ interactions with GenAI differ from those of other health care professionals in terms of workflow, care responsibilities, and risk exposure. Nurses are often responsible for continuous bedside assessment, patient education, nursing documentation, handover communication, care coordination, and early identification of patient deterioration. When GenAI is used to support these tasks, nurses must be able to verify generated content, identify hallucinations or biased recommendations, protect patient data, and determine whether outputs are appropriate for individual patient contexts. These competencies are closely linked to nursing judgment, professional accountability, and patient safety. This gap underscores the need for a GenAI literacy assessment tool specifically designed for nurses.

Therefore, this study aimed to develop and validate the GenAILS. A psychometrically sound assessment tool grounded in the technical and ethical context of nursing was established, providing a foundation for future clinical application, education, and policy development.

## Methods

### Research Design

This 2-phase cross-sectional study aimed to develop and validate the GenAILS. To ensure linguistic and cultural relevance for clinical nurses in Taiwan, the initial item pool and final scale were developed and administered in traditional Chinese. The English item descriptions presented in the tables were author-prepared translations for reporting purposes only and were not used for data collection. No formal forward-backward translation or psychometric validation of an English version was conducted in this study.

In phase one, items were generated through literature-based conceptualization and expert review, followed by content validity evaluation and item refinement (items with a content validity index <0.78 were revised or deleted). In phase 2, the scale’s psychometric properties were examined through item analysis, reliability testing (internal consistency and split-half reliability), criterion-related validity, and construct validity using exploratory factor analysis (EFA; factor loading ≥0.60) and confirmatory factor analysis (CFA).

This study was reported with reference to the relevant items of the Consensus-Based Standards for the Selection of Health Measurement Instruments (COSMIN) reporting guideline, version 2.0 [23], and the CHERRIES (Checklist for Reporting Results of Internet E-Surveys) [24]. Because the GenAILS is a self-report instrument assessing nurses’ perceived GenAI literacy and professional competencies, the COSMIN guidelines were not fully applicable to this study. Nevertheless, the reporting items on content validity, structural validity, internal consistency, reliability, and criterion-related validity served as important references for improving the reporting quality of this study.

### Conceptualization

The initial phase involved rigorous construct conceptualization through a comprehensive literature review. Clearly articulating the construct composition and using a theoretical framework help ensure the clarity and validity of the scale [25]. The theoretical framework, research objectives, and methodological procedures were systematically established based on existing evidence. Based on a comprehensive review identifying attributes across nursing, education, and AI literacy, the generative artificial intelligence literacy for nurses framework was conceptualized, comprising 6 distinct dimensions.

First, responsible use underscores the necessity for users to possess evaluative capabilities and a commitment to responsible application. It entails avoiding misuse or over-reliance on tools. At the technical level, users must effectively use GenAI to enhance efficiency, creativity, and problem-solving across diverse contexts. It also involves identifying AI-generated content to ensure source credibility and authenticity, thereby preventing the circulation of fabricated or erroneous information. Users must judge the appropriateness of AI tools and apply effective prompting to guide systems toward specific goals. Accordingly, Responsible Use includes selecting suitable GenAI tools for specific tasks and applying prompt engineering strategies that align outputs with user intent [1]. Furthermore, it requires responsible application of AI principles during operation to mitigate foreseeable risks [26]. Within nursing practice and education, this dimension emphasizes adherence to responsible use principles that maintain human oversight in decision-making, ensuring that GenAI functions as an assistive resource rather than a substitute for professional judgment [1,9,10].

Second, updated competencies highlight the necessity of continuous learning in response to rapid technological advancement. Continuous learning forms the foundation of literacy. Users must proactively update their technical knowledge, operational skills, and ethical understanding to respond to the evolving AI landscape. Given the diversity of available tools, users should become familiar with various functionalities and select appropriate tools for specific applications through ongoing specialized training. Because GenAI spans multiple domains, literacy is a dynamic process that evolves with technological advancement rather than a static state. Operationally, Updated Competencies include proactively learning new tools, functions, and relevant regulations as technology evolves, thereby maintaining current literacy and supporting responsible GenAI use [1].

Third, risk identification focuses on recognizing and anticipating GenAI-related risks in health care. AI algorithms may harbor biases that contribute to unequal health care outcomes. When using GenAI, users must consider risks such as inaccurate information, hallucinations, and data security vulnerabilities [27]. Because medical data are highly sensitive (eg, patient health records), robust protection mechanisms are required to prevent security breaches [28]. Explicit monitoring policies and user education are therefore essential to manage and mitigate risks associated with GenAI applications in health care [27]. Operationally, risk identification includes vigilance regarding security threats and the potential for GenAI to be exploited for deception, including the production of deepfake text, images, or audio. Furthermore, it requires identifying both the strengths and limitations of GenAI, particularly the risk of inaccurate outputs and plausible yet clinically unsafe hallucinations [1].

Fourth, fundamental knowledge encompasses the foundational understanding required to comprehend AI’s operational mechanisms, serving as a prerequisite for advanced learning [14]. Learners should understand GenAI as a statistical model trained on massive datasets capable of generating new multimodal content, such as text, images, and code [6,7]. This dimension requires distinguishing GenAI from traditional search engines and recognizing that GenAI creates content, whereas search engines retrieve existing data. Furthermore, users should differentiate its generative nature from the discriminative focus (recognition and classification) characteristic of other AI systems [1,7].

Fifth, critical evaluation addresses the essential capacity to appraise GenAI outputs with clinical rigor. As GenAI becomes increasingly embedded in medical and nursing education, its influence extends beyond academic support to professional practice, where appropriate and critical application is paramount [21]. With ongoing technological advancement, curriculum designers and learners must prioritize critical assessment of generated content to ensure safe use [9]. Although GenAI may augment human capabilities, excessive reliance may undermine critical thinking and problem-solving. Moreover, the risks of misuse or malfunction increase as AI systems become more autonomous, potentially threatening patient safety. A structured framework for critical thinking and oversight is therefore necessary to ensure safe and accountable use [28]. Integrating critical and creative thinking into AI education is a key strategy for strengthening learner literacy and preparedness [26]. Users should critically evaluate the quality, accuracy, bias, and appropriateness of GenAI-generated content, particularly when outputs inform clinical decisions or educational activities. Users must also verify whether generated content aligns with specific clinical needs. They should adjust GenAI use accordingly to achieve intended outcomes while maintaining professional standards [1,9].

Sixth, ethics and law address the ethical and legal responsibilities associated with AI integration. As AI increasingly influences decisions traditionally made by humans, reassessing ethical responsibilities, privacy, and moral standards is imperative. This evolution necessitates a collaborative partnership between humans and AI [29]. Users must understand the ethical implications of AI models and their outputs. They must also be familiar with relevant legal and regulatory frameworks to ensure compliance. From an educational perspective, institutions should establish systematic training to prepare the workforce to address issues such as privacy protection, bias mitigation, equity, and intellectual property, thereby fostering responsible and lawful GenAI use [1]. This dimension aligns with the World Health Organization [30] guidance on AI ethics in health. That guidance emphasizes six core principles: (1) protect autonomy; (2) promote human well-being, safety, and the public interest; (3) ensure transparency, explainability, and intelligibility; (4) foster responsibility and accountability; (5) ensure inclusiveness and equity; and (6) promote responsive and sustainable AI. Operationally, Ethics and Law involves awareness of ethical concerns such as privacy, fairness, intellectual property, and data bias [1,9,10]. Furthermore, it includes recognizing potential AI misuse in producing forged or misleading information. It also requires upholding data privacy and transparency in AI-driven decision-making within highly automated health care environments. Clear accountability, human oversight, and governance mechanisms are required to manage risks and unintended consequences [1,10].

### Item Generation

Regarding item pool generation, two primary strategies can be distinguished: the deductive (top-down) approach, which derives items from construct definitions, literature, and existing scales, and the inductive (bottom-up) approach, which relies on qualitative data such as focus groups or interviews [31]. The deductive (top-down) approach was adopted. Items were generated based on the operational definitions of the six identified dimensions, a systematic review of relevant literature, and analysis of existing AI literacy instruments.

The item generation process involved 3 sequential steps. First, grounded in the core construct of “AI Literacy,” relevant dimensions and competency indicators were extracted from existing scales [32,33], and evidence from the literature review was synthesized to develop an initial pool of 36 items (see Conceptualization section). Second, an expert committee comprising 6 specialists in nursing education and clinical practice conducted an open-ended review. The committee recommended refining the focus from general AI literacy to GenAI literacy to better address emerging clinical needs and provided guidance for revising the dimensions and items to improve clarity and clinical appropriateness. Third, the construct was subsequently refined to “GenAI Literacy,” and an expanded and revised pool of 50 items across 6 dimensions was developed. To ensure semantic clarity and minimize respondent burden, item construction adhered to scale development principles and avoided double-barreled questions, ambiguous wording, and negative phrasing [31].

Content validity: to establish content validity, the 50-item pool was evaluated by a panel of 5 experts, including specialists in psychometrics, clinical nursing education, and AI. Each item’s relevance, clarity, and necessity were assessed using the item-level content validity index (I-CVI) and scale content validity index based on the average method. Experts rated items on a 4-point Likert scale. Items were retained if they met the acceptance criteria of I-CVI ≥0.78 and scale content validity index based on the average method ≥0.90 [34]. Items flagged for revision were modified based on qualitative expert feedback to enhance validity.

Face validity (cognitive interviewing): following content validity assessment, face validity was examined to evaluate readability and respondent comprehension. The cognitive interviewing method was adopted, and seven registered nurses participated in in-depth interviews to explore their understanding of the items. This sample size aligned with the recommendation of 5-15 participants for cognitive interviewing [35]. Participants provided feedback on item ambiguity, logical flow, and estimated completion time. Necessary revisions were subsequently made to finalize the scale items.

### Psychometric Evaluation: Sample and Data Collection

This cross-sectional study collected data through a web-based questionnaire hosted on SurveyCake using convenience sampling between June 2025 and October 2025. Data collection was completed in October 2025, and the dataset was locked before data screening and statistical analysis.

Participants were recruited through social media posts, email invitations distributed via the researchers’ professional and personal networks, and direct recruitment of clinical nurses in several hospitals after permission had been obtained from nurse managers. The recruitment information included the study purpose, eligibility criteria, voluntary nature of participation, and survey link. Eligible participants were current registered nurses working in hospitals, able to complete the online questionnaire independently, and able to read and understand traditional Chinese. The survey landing page clearly stated these eligibility criteria, and participants were required to confirm their eligibility before proceeding. Electronic informed consent was obtained by selecting the online “Consent to Participate” option.

To further verify participants’ nursing status, a nursing professional knowledge screening item was embedded in the questionnaire before the GenAILS items. This item assessed basic clinical knowledge related to indwelling urinary catheter care and was designed to help distinguish eligible registered nurses from non-nursing respondents, as it required knowledge typically acquired through nursing education and clinical training. Responses that failed to meet the eligibility criteria or showed inconsistent professional information were excluded during data screening. Previous studies have noted that insufficient effort in responding to online surveys can compromise psychometric properties, resulting in reduced reliability, increased measurement error, attenuated correlations, and biased factor-analytic results [36]. Thus, a rigorous multi-stage quality control strategy was implemented to protect data integrity.

Data quality was controlled through multiple procedures. Invariant responding was screened by flagging respondents whose continuous response pattern (eg, repeatedly selecting the same option) exceeded half the total scale length, in accordance with previous recommendations [37]. Speeding was assessed by applying a minimum time threshold; responses averaging <2 seconds per item were classified as overly rapid and excluded [36,38]. The survey also incorporated instructed-response items (requiring selection of a specified option) and bogus items (inquiring about impossible events) to detect inconsistent or random responding [36]. Professional identity was also verified. Responses that failed these logic checks, failed the nursing professional knowledge screening item, or violated instructions were deemed invalid. Technical safeguards, including automated bot-detection algorithms, were applied to identify and exclude computer-generated responses. To prevent multiple submissions (duplicate submissions), participants were required to bind a unique email address to their response, restricting participation to one submission per account. A quality assurance statement was displayed on the survey landing page to enhance attentiveness and data accuracy. Participants were informed that responses would undergo systematic quality checks for logical consistency, response latency, and adherence to instructions, and that failure to meet these criteria would result in exclusion and ineligibility for the incentive (gift voucher).

Initially, 1313 questionnaires were collected. Of these, 191 invalid responses were excluded following quality control procedures, including failure to meet inclusion criteria (n=13), refusal to participate (n=13), response invariability (n=91), failure on instructed-response items (n=25), response time below the minimum threshold (n=11), and duplicate email submissions (n=38). Consequently, 1122 valid responses were analyzed.

### Data Analysis

Data were encoded and analyzed using SPSS Statistics (IBM Corp; version 29.0) and LISREL (version 8.8; Scientific Software International). The statistical analysis included item analysis, construct validity testing, criterion-related validity assessment, and reliability assessment.

Step 1 was item analysis. Descriptive statistics, including mean, variance, skewness, and kurtosis, were calculated to evaluate the distribution of item responses. Item discrimination was assessed via two methods: (1) corrected item-total correlation: items with a correlation coefficient <0.30 were considered for deletion [31,39]; and (2) extreme-group comparison: based on Kelley [40] method, the sample was divided into high (top 27%) and low-scoring (bottom 27%) groups. Independent-samples *t* tests were conducted to examine statistically significant differences between the groups. Additionally, Cronbach α was examined if an item was deleted; items were removed if their deletion significantly improved the overall reliability coefficient.

Step 2 was assessing construct validity via EFA and CFA. A random splitting method was used to divide the sample into two independent subsets (subsamples 1 and 2) for EFA and CFA. According to Gorsuch recommendation for factor analysis, an adequate sample should include at least 5 participants per item and an absolute minimum of 100 participants, regardless of the number of items [41]. In this study, the EFA subsample included 561 participants for the initial 46-item pool, and the independent CFA subsample included 561 participants for the final 24-item model. Therefore, both subsamples exceeded the recommended subject-to-item ratio and minimum absolute sample size criteria. EFA was conducted to explore the underlying factor structure [42,43]. Data suitability was assessed using the Kaiser-Meyer-Olkin (KMO) test and Bartlett test of sphericity. EFA was conducted using principal axis factoring (PAF) with Promax rotation. Items were retained if they had a factor loading of ≥0.60. CFA was conducted to verify the factor structure [42]. Model fit was evaluated using the following indices: root-mean-square error of approximation (RMSEA) ≤0.08; standardized root-mean-square residual (SRMR) ≤0.08; comparative fit index (CFI) ≥0.90; goodness-of-fit index (GFI) ≥0.90; adjusted goodness-of-fit index (AGFI) ≥0.85; nonnormed fit index (NNFI) ≥0.90; parsimony normed fit index (PNFI) ≥0.80 [44-46]. Convergent validity was assessed based on composite reliability (CR) >0.70 and average variance extracted (AVE) >0.36 (preferably >0.50) [47,48].

Discriminant validity was assessed using the heterotrait-monotrait ratio (HTMT). Traditional methods for assessing discriminant validity include the Fornell-Larcker criterion and cross-loadings; however, their sensitivity in detecting insufficient discriminant validity is relatively limited. Previous simulation studies have shown that the Fornell-Larcker criterion detects a lack of discriminant validity in more than 50% of simulation runs only under conditions with highly heterogeneous indicator loading patterns and sample sizes of 500 or less. In contrast, when indicator loadings are more homogeneous, the sensitivity of the Fornell-Larcker criterion decreases substantially, particularly when the AVE is low. HTMT is calculated as the ratio of the average correlations between indicators across different constructs to the geometric mean of the average correlations among indicators within the same construct, and is used to determine whether latent constructs are sufficiently distinct from one another. According to the more stringent criterion, HTMT values below 0.85 were considered evidence of adequate discriminant validity [49]. Criterion-related validity was assessed by calculating Pearson correlation coefficient between the GenAILS and the Short Form Meta-AI Literacy Scale [50], with *r*>0.50 interpreted as indicating a moderate-to-strong association [51,52].

Reliability was assessed using multiple indicators, including (1) internal consistency (Cronbach α): Cronbach α was calculated to evaluate the internal consistency of items within each subscale [53]. A coefficient >0.70 was established as the threshold for acceptable reliability [54,55]; (2) McDonald *ω*: this was used due to the limitations of Cronbach α, which assumed tau-equivalence (ie, equal factor loadings for all items). Since McDonald *ω* does not require this assumption and accounts for varying item contributions to the construct, it was considered a more accurate estimate of true reliability [31,55]. Thus, this study also computed McDonald *ω*; (3) Split-half reliability: to further verify internal consistency, split-half reliability was examined by dividing the scale items into odd and even subsets [55]. Subsequently, the Spearman-Brown prediction formula was applied to correct for the shortened length and estimate the scale’s reliability.

### Ethical Considerations

This study was approved by the Institutional Review Board of the MacKay Memorial Hospital (approval number 25MMHIS007e). All participants were informed of the study purpose, procedures, and their rights, including the right to withdraw at any time without penalty, in accordance with the Declaration of Helsinki and its subsequent amendments. Informed consent was obtained digitally; participants were required to check the “Consent to Participate” box on the survey landing page before accessing the questionnaire. All data were de-identified and stored securely to protect participant privacy.

## Results

### Content Validity and Face Validity

Quantitative assessment of content validity demonstrated excellent expert consensus. The overall scale-level content validity index based on the average method was 0.92 (relevance=0.94; clarity=0.86; necessity=0.94). Based on qualitative feedback from external experts, minor semantic revisions were made to enhance item clarity and precision and were subsequently verified by two additional experts. Consequently, the initial 50-item pool was refined to a 46-item draft scale for subsequent psychometric evaluation (Multimedia Appendix 1).

Face validity was established via cognitive interviewing with seven registered nurses who met the inclusion criteria. Feedback indicated that the items possessed high semantic clarity and clinical relevance, with no significant logical conflicts reported. Consequently, no items were deleted, and the 46-item structure was retained.

### Psychometric Evaluation: Participants’ Characteristics

The final sample comprised 1122 registered nurses (Table 1). Participants’ mean age was 34.7 (SD 7.8) years. Most participants were female (876/1122, 78.1%). Regarding practice setting, participants predominantly worked in medical centers (469/1122, 41.8%) and regional hospitals (435/1122, 38.8%). Primary clinical units included surgical wards (205/1122, 18.3%), internal medicine wards (199/1122, 17.7%), and intensive care units (167/1122, 14.9%). Regarding professional experience, most participants had 6-10 years (316/1122, 28.2%) or 11-15 years (289/1122, 25.8%) of nursing experience. Most participants were registered nurses (1000/1122, 89.1%), with a smaller proportion holding administrative or supervisory positions (122/1122, 10.9%).

**Table 1:** Participant characteristics.

Baseline characteristics		Total sample (N=1122), n (%)	Subsample 1^a^ (n=561), n (%)	Subsample 2^a^ (n=561), n (%)
**Age (years)**				
	20-30	388 (34.6)	193 (34.4)	195 (34.8)
	31-40	476 (42.4)	238 (42.4)	238 (42.4)
	41-50	213 (19)	111 (19.8)	102 (18.2)
	≥51	45 (4)	19 (3.4)	26 (4.6)
**Sex**				
	Male	226 (20.1)	115 (20.5)	111 (19.8)
	Female	876 (78.1)	433 (77.2)	443 (79)
	Prefer not to disclose	20 (1.8)	13 (2.3)	7 (1.2)
**Highest level of education**				
	Junior college (associate’s degree)	89 (7.9)	43 (7.7)	46 (8.2)
	Two-year technical program	263 (23.4)	129 (23)	134 (23.9)
	Four-year technical program	166 (14.8)	80 (14.3)	86 (15.3)
	Bachelor’s degree	493 (43.9)	250 (44.6)	243 (43.3)
	Master’s degree	109 (9.7)	59 (10.5)	50 (8.9)
	Doctoral degree	2 (0.2)	0 (0)	2 (0.4)
**Department**				
	Internal medicine ward	199 (17.7)	101 (18)	98 (17.5)
	Surgical ward	205 (18.3)	105 (18.7)	100 (17.8)
	Emergency department	130 (11.6)	58 (10.3)	72 (12.8)
	Intensive care unit	167 (14.9)	89 (15.9)	78 (13.9)
	Psychiatric ward	38 (3.4)	19 (3.4)	19 (3.4)
	Obstetrics and gynecology ward	54 (4.8)	29 (5.2)	25 (4.5)
	Pediatrics ward	54 (4.8)	27 (4.8)	27 (4.8)
	Hematology and oncology ward	54 (4.8)	26 (4.6)	28 (5)
	Palliative care ward	17 (1.5)	8 (1.4)	9 (1.6)
	Home care	17 (1.5)	8 (1.4)	9 (1.6)
	Hemodialysis unit	99 (8.8)	44 (7.8)	55 (9.8)
	Operating room	26 (2.3)	15 (2.7)	11 (2)
	Anesthesiology	18 (1.6)	6 (1.1)	12 (2.1)
	Long-term care	10 (0.9)	8 (1.4)	2 (0.4)
	Administrative unit	33 (2.9)	18 (3.2)	15 (2.7)
	Others	1 (0.1)	0 (0)	1 (0.2)
**Hospital level**				
	Medical center	469 (41.8)	239 (42.6)	230 (41)
	Regional hospital	435 (38.8)	216 (38.5)	219 (39)
	District hospital	218 (19.4)	106 (18.9)	112 (20)
**Years of nursing experience**				
	0-2	57 (5.1)	28 (5)	29 (5.2)
	3-5	138 (12.3)	75 (13.4)	63 (11.2)
	6-10	316 (28.2)	156 (27.8)	160 (28.5)
	11-15	289 (25.8)	142 (25.3)	147 (26.2)
	16-20	159 (14.2)	84 (15)	75 (13.4)
	≥21	163 (14.5)	76 (13.5)	87 (15.5)
**Current nursing position**				
	Registered nurse	1000 (89.1)	496 (88.4)	504 (89.8)
	Assistant head nurse	52 (4.6)	27 (4.8)	25 (4.5)
	Head nurse	61 (5.4)	32 (5.7)	29 (5.2)
	Supervisor	9 (0.8)	6 (1.1)	3 (0.5)

^a^Random splitting was used to create subsample 1 for exploratory factor analysis and subsample 2 for confirmatory factor analysis.

### Item Analysis

Item analysis was conducted on the initial 46-item scale to evaluate its suitability for factor analysis. Descriptive statistics indicated that item means ranged from 3.6 to 4.1 (SDs ranging from 0.67-1.01), with variances between 0.45 and 1.03. Data distribution was slightly negatively skewed, with skewness and kurtosis values ranging from –0.64 to –0.16 and –0.45 to 0.86, respectively. All absolute values for skewness and kurtosis were <1, indicating that the data did not deviate significantly from a normal distribution. Item discrimination was assessed using the extreme-group comparison method. Independent-samples *t* tests revealed statistically significant differences for all 46 items between the high- and low-scoring groups (*P*<.001), demonstrating excellent discriminatory power. Furthermore, corrected item-total correlations ranged from 0.426 to 0.639 and consistently exceeded the retention threshold of 0.30. Internal consistency analysis revealed that the overall Cronbach α was 0.95; excluding any single item resulted in values ranging from 0.95 to 0.96. Since removing items did not improve reliability, all 46 items were retained for subsequent EFA (Multimedia Appendix 2).

### Exploratory Factor Analysis (Subsample 1)

EFA was conducted on subsample 1 (n=561) to examine the scale’s latent factor structure. The KMO measure of sampling adequacy was 0.93, and Bartlett’s test of sphericity was statistically significant (χ^2^=5971.76; *P*<.001), confirming the suitability of the data for factor analysis. PAF with promax rotation was used because the underlying dimensions of GenAI literacy were theoretically expected to be correlated. The retention criterion for factor loadings was set at ≥0.60 to ensure robust item-factor relationships; this stricter cut-off was adopted to retain only items with high explanatory power for this newly developed instrument [56]. The analysis yielded a 6-factor solution accounting for 53.1% of the total variance. Although only the first two eigenvalues exceeded 1 (8.50 and 1.28), the six-factor structure was retained based on the a priori conceptual framework, theoretical interpretability, and the observed factor loading pattern rather than the Kaiser criterion alone. Factor loadings for the retained items ranged from 0.63 to 0.81. The final EFA reduced the 46-item draft to a 24-item scale (Table 2). Specifically, 24 items were retained across six dimensions: factor 1, responsible use (5 items); factor 2, updated competencies (4 items); factor 3, risk identification (4 items); factor 4, fundamental knowledge (4 items); factor 5, critical evaluation (4 items); and factor 6, ethics and law (3 items).

**Table 2:** Exploratory factor analysis item loadings, subsample 1 (n=561).

Item number	Item description	Factor loading											
		Factor 1^a^		Factor 2^b^		Factor 3^c^		Factor 4^d^		Factor 5^e^		Factor 6^f^	
11	I can use GenAI^g^ to assist in creating nursing educational materials.	0.65		—^h^		—		—		—		—	
12	I can use GenAI to assist in generating administrative reports.	0.76		—		—		—		—		—	
13	I can use GenAI to design individualized health education content based on the patient’s condition.	0.67		—		—		—		—		—	
14	I can use GenAI to provide specific care recommendations as a clinical reference.	0.81		—		—		—		—		—	
15	I can use GenAI to generate clinical data charts to assist in formulating patient care plans.	0.69		—		—		—		—		—	
43	I actively keep up with rapid changes in the clinical application of GenAI.	—		0.72		—		—		—		—	
44	I continuously update my competencies in applying GenAI.	—		0.64		—		—		—		—	
45	I proactively seek assistance when encountering difficulties in using GenAI.	—		0.67		—		—		—		—	
46	I actively learn new applications and innovative methods of GenAI.	—		0.75		—		—		—		—	
36	I can identify hallucinations in data generated by GenAI.	—		—		0.66		—		—		—	
39	I can identify risks resulting from erroneous reasoning by GenAI.	—		—		0.72		—		—		—	
40	I can identify potential inaccuracies or lack of representativeness in the training data used by GenAI.	—		—		0.77		—		—		—	
41	I can identify the applicability and potential biases of GenAI across different population groups.	—		—		0.64		—		—		—	
2	I understand the differences between GenAI and discriminative AI in clinical applications.	—		—		—		0.64		—		—	
3	I understand the functional differences among common GenAI tools.	—		—		—		0.71		—		—	
4	I understand how GenAI undergoes model training using massive datasets.	—		—		—		0.65		—		—	
5	I understand how GenAI generates responses through prompts.	—		—		—		0.73		—		—	
18	I can critically evaluate whether the nursing guidance produced by GenAI meets the needs of individual patients.	—		—		—		—		0.80		—	
19	I can critically evaluate the clinical applicability of nursing documentation generated by GenAI.	—		—		—		—		0.67		—	
21	I can make critical decisions when GenAI outputs conflict with clinical professional judgment.	—		—		—		—		0.71		—	
23	I can determine whether to modify or discard GenAI recommendations based on the clinical context.	—		—		—		—		0.67		—	
33	When GenAI is involved in care decisions, I comply with legal regulations regarding data processing in clinical settings.	—		—		—		—		—		0.64	
34	When GenAI is involved in care decisions, I understand that health care professionals bear ultimate decision-making responsibility and legal liability.	—		—		—		—		—		0.71	
35	When GenAI is involved in care decisions, I can identify potential ethical controversies in generated content.	—		—		—		—		—		0.63	
Eigenvalues			8.5		1.28		0.97		0.88		0.69		0.48
Explained variance (%)			35.3		5.3		4		3.7		2.9		2
Cumulative variance (%)			35.3		40.6		44.6		48.2		51.1		53.1

^a^Factor 1: responsible use.

^b^Factor 2: updated competencies.

^c^Factor 3: risk identification.

^d^Factor 4: fundamental knowledge.

^e^Factor 5: critical evaluation.

^f^Factor 6: ethics and law.

^g^GenAI: generative artificial intelligence.

^h^Not applicable.

### Confirmatory Factor Analysis (Subsample 2)

**Figure 1:**
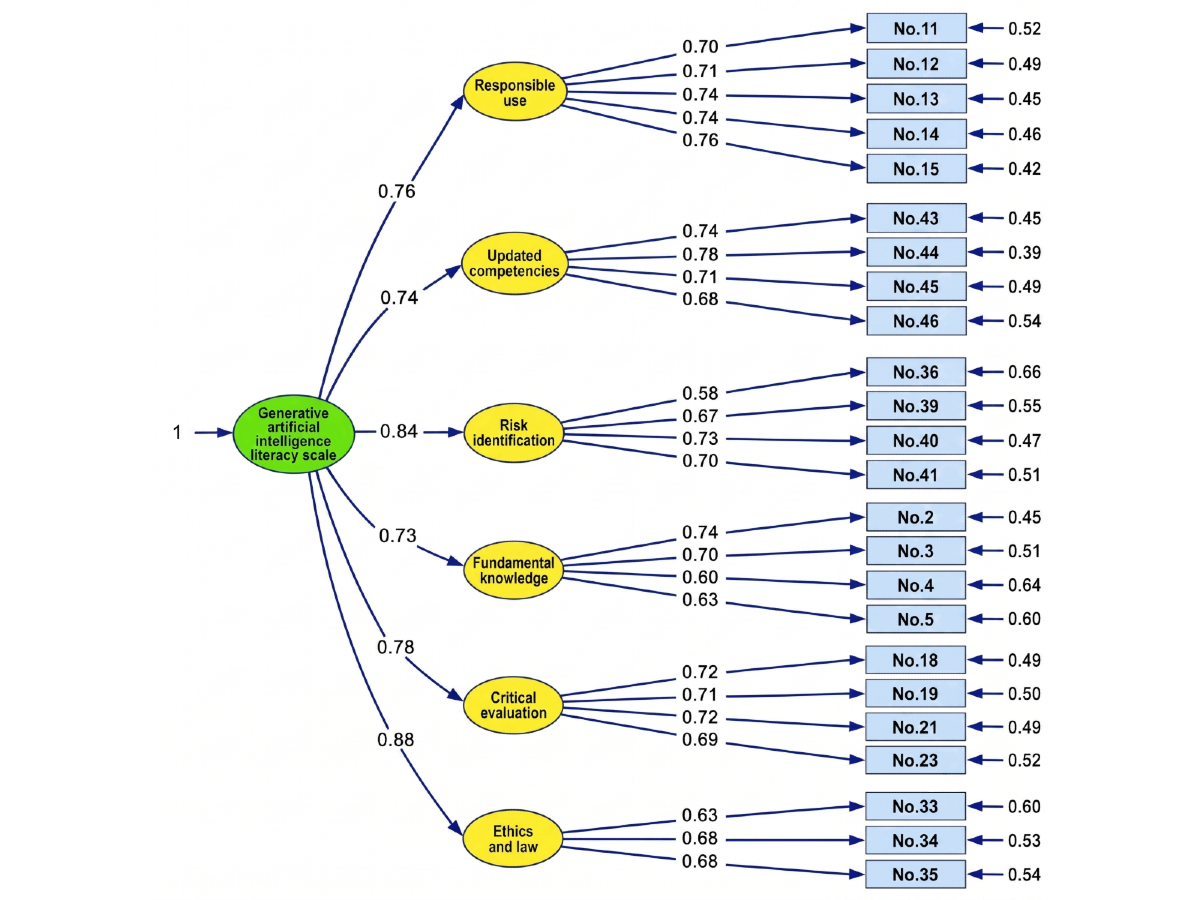
Second-order confirmatory factor analysis path diagram of the Generative Artificial Intelligence Literacy Scale for Nurses, subsample 2 (n=561).

To verify the scale’s factor structure and construct validity, CFA was conducted using subsample 2 (n=561) based on the 24-item, six-factor structure identified through the EFA. A first-order CFA model was first tested, followed by a second-order CFA model to examine whether the six first-order dimensions could be represented by a higher-order GenAI literacy construct (Figure 1).

All 24 items loaded statistically significantly on their respective first-order dimensions, with standardized factor loadings ranging from 0.58 to 0.78. The CR for the six dimensions ranged from 0.70 to 0.85, meeting the recommended threshold of 0.70 [57]. The AVE values ranged from 0.44 to 0.53 (Table 3). The first-order model demonstrated excellent fit: RMSEA=0.035 (90% CI 0.029-0.041), SRMR=0.032, CFI=0.99, GFI=0.94, AGFI=0.93, NNFI=0.99, and PNFI=0.84.

**Table 3:** Item standardized loadings from the confirmatory factor analysis with subsample 2 (n=561).

Dimensions and Item number		CFA^a^ (factor loading)	CR^b^		AVE^c^		Cronbach α (95% CI)		McDonald ω	
**Responsible use**				0.85		0.53		0.85 (0.84-0.86)		0.85
	No.11	0.70								
	No.12	0.71								
	No.13	0.74								
	No.14	0.74								
	No.15	0.76								
**Updated competencies**				0.82		0.53		0.80 (0.79-0.82)		0.81
	No.43	0.74								
	No.44	0.78								
	No.45	0.71								
	No.46	0.68								
**Risk identification**				0.77		0.45		0.78 (0.75-0.80)		0.78
	No.36	0.58								
	No.39	0.67								
	No.40	0.73								
	No.41	0.70								
**Fundamental knowledge**				0.76		0.45		0.77 (0.75-0.79)		0.77
	No.2	0.74								
	No.3	0.70								
	No.4	0.60								
	No.5	0.63								
**Critical evaluation**				0.80		0.50		0.81 (0.80-0.83)		0.81
	No.18	0.72								
	No.19	0.71								
	No.21	0.72								
	No.23	0.69								
**Ethics and law**				0.70		0.44		0.73 (0.70-0.75)		0.73
	No.33	0.63								
	No.34	0.68								
	No.35	0.68								
**Second-order model**				0.91		0.63		0.92 (0.91-0.92)		0.92
	Factor (1)	0.76								
	Factor (2)	0.74								
	Factor (3)	0.84								
	Factor (4)	0.73								
	Factor (5)	0.78								
	Factor (6)	0.88								

^a^CFA: confirmatory factor analysis.

^b^CR: composite reliability.

^c^AVE: average variance extracted.

Statistically significant positive correlations were observed among the 6 first-order dimensions (*P*<.001), supporting specification of the higher-order model. In the second-order CFA, the 6 dimensions loaded significantly onto the overarching GenAI Literacy construct, with standardized second-order loadings ranging from 0.73 to 0.88. The second-order construct demonstrated strong reliability and convergent validity, with a CR of 0.91 and an AVE of 0.63. The second-order measurement model demonstrated excellent fit to the data. Fit indices were RMSEA=0.039 (90% CI 0.033-0.045), SRMR=0.040, CFI=0.99, GFI=0.94, AGFI=0.92, NNFI=0.99, and PNFI=0.87. These results supported the structural validity and hierarchical structure of the GenAILS (Table 4).

**Table 4:** Model fit indices.

Fit index	First-order model	Second-order model
RMSEA^a^ (90% CI)	0.035 (0.029-0.041)	0.039 (0.033-0.045)
SRMR^b^	0.032	0.040
CFI^c^	0.99	0.99
GFI^d^	0.94	0.94
AGFI^e^	0.93	0.92
NNFI^f^	0.99	0.99
PNFI^g^	0.84	0.87

^a^RMSEA: root-mean-square error of approximation.

^b^SRMR: standardized root-mean-square residual.

^c^CFI: comparative fit index.

^d^GFI: goodness-of-fit index.

^e^AGFI: adjusted goodness-of-fit index.

^f^NNFI: nonnormed fit index.

^g^PNFI: parsimony normed fit index.

### Reliability

The GenAILS’s internal consistency was evaluated using Cronbach α and McDonald *ω* coefficients. The overall scale demonstrated excellent reliability, with a Cronbach α of 0.92 (95% CI 0.91-0.92) and McDonald *ω* of 0.92. For the 6 individual dimensions, Cronbach α and McDonald *ω* coefficients ranged from 0.73-0.85, all exceeding the acceptable threshold of 0.70. Additionally, split-half reliability was assessed by dividing the scale items into odd- and even-numbered subsets (Table 3). The Spearman-Brown corrected coefficient was 0.81, confirming high internal consistency.

### Correlations Among Constructs

All inter-factor correlations were positive and moderate-to-strong (approximately *r*=0.54-0.74) and statistically significant (*P*<.001), indicating that the dimensions were related but not redundant. The overall scale showed strong correlations between the total score and each subscale (r=0.73-0.88; Table 5).

**Table 5:** The correlations among the first-order factors (N=1122).

Factors	1	2	3	4	5	6
1. Responsible use	1	—^a^	—	—	—	—
2. Updated competencies	0.56	—	—	—	—	—
3. Risk identification	0.60	0.58	—	—	—	—
4. Fundamental knowledge	0.64	0.62	0.66	—	—	—
5. Critical evaluation	0.55	0.54	0.57	0.61	—	—
6. Ethics and law	0.67	0.65	0.69	0.74	0.64	—
Overall scale	0.76	0.74	0.78	0.84	0.73	0.88

^a^not applicable.

### HTMT

The HTMT values among the 6 GenAILS dimensions ranged from 0.53 to 0.83 (Table 6). All values were below the conservative threshold of 0.85, supporting adequate discriminant validity among the six dimensions. The highest HTMT value was observed between Risk Identification and Ethics and Law (HTMT=0.83), suggesting that these two dimensions were closely related but still empirically distinguishable. Because this value approached the conservative threshold, future studies should monitor this pair for potential redundancy across samples with different demographic and professional characteristics. The lowest HTMT value was observed between Updated Competencies and Fundamental Knowledge (HTMT=0.53). These findings indicate that the six dimensions of the GenAILS represent related but distinct components of nurses’ GenAI literacy.

**Table 6:** Heterotrait-monotrait ratio.

Factors	1	2	3	4	5	6
1.Responsible use	—^a^	—	—	—	—	—
2.Updated competencies	0.56	—	—	—	—	—
3. Risk identification	0.56	0.62	—	—	—	—
4. Fundamental knowledge	0.69	0.53	0.57	—	—	—
5. Critical evaluation	0.65	0.56	0.69	0.56	—	—
6. Ethics and law	0.60	0.69	0.83	0.61	0.63	—

^a^Not applicable.

### Criterion-Related Validity

Criterion-related validity was evaluated as concurrent validity by examining the correlation between GenAILS scores and the Short Form Meta-AI Literacy Scale [50], a previously validated measure of general AI literacy-related competencies. This scale was selected as a conceptually related external criterion because validated GenAI literacy instruments specifically tailored to clinical nursing contexts remain limited. The criterion measure demonstrated acceptable reliability (Cronbach α=0.80; McDonald ω=0.75), with the modest difference between α and ω suggesting possible heterogeneous item contributions or slight departures from tau-equivalence. Pearson’s correlation analysis showed a statistically significant moderate positive association between the GenAILS and the criterion measure (*r*=0.57; *P*<.001), supporting the criterion-related validity of the GenAILS. Because the criterion measure assesses general AI literacy rather than GenAI-specific nursing literacy, this association should be interpreted as evidence of external convergence with a related construct rather than evidence that the 2 instruments measure identical competencies.

## Discussion

### Principal Findings and Psychometric Performance

This study developed and psychometrically validated the GenAILS (Multimedia Appendix 3), a nurse-specific self-report instrument for assessing self-perceived GenAI literacy. The final 24-item scale comprises six dimensions: Responsible Use, Updated Competencies, Risk Identification, Fundamental Knowledge, Critical Evaluation, and Ethics and Law. Each item is rated on a 5-point Likert scale ranging from 1 (strongly disagree) to 5 (strongly agree). Scores can be calculated at both the subscale and total-scale levels.

Evidence of content validity was supported by expert review (scale-level content validity index based on the average method=0.92). Item analysis demonstrated acceptable distributional properties (|skewness| and |kurtosis| <1) and strong discrimination using the extreme-group comparison method (high- and low-scoring groups 27%; all *P*<.001). Construct validity was supported through EFA using a stringent retention criterion (factor loadings ≥0.60) and CFA. Specifically, in the EFA stage, the six-factor solution explained 53.1% of the variance, which was considered reasonable for a multidimensional self-report construct because PAF, as a common factor approach, focuses on common variance rather than unique or error variance [56]. Convergent validity was supported by first-order CFA results, with CR values ranging from 0.70 to 0.85 and AVE values ranging from 0.44 to 0.53. Building upon these exploratory findings, the second-order model further supported the overarching GenAI literacy construct, with CR=0.91, AVE=0.63, and excellent model fit.

Discriminant validity was also supported by HTMT values ranging from 0.53 to 0.83, all below the conservative threshold of 0.85. Criterion-related validity was supported by a statistically significant moderate correlation with the Short Form Meta-AI Literacy Scale (r=0.57, *P*<.001). Reliability was strong across subscales, with Cronbach α and McDonald ω values ranging from 0.73 to 0.85, and was excellent for the total scale (Cronbach α=0.92; McDonald ω=0.92). These findings indicate that the GenAILS is a psychometrically sound instrument for assessing GenAI literacy among clinical nurses.

### Comparison With Existing Instruments

To situate the unique contribution of the GenAILS, we compared it with existing instruments developed for related constructs and different target populations. The Medical Artificial Intelligence Readiness Scale for Medical Students [58] evaluates medical students’ perceived readiness for AI technologies and applications. The Teacher Artificial Intelligence Competence Self-Efficacy Scale [59] and Readiness for Artificial Intelligence Applications Scale [60] target teachers. The Artificial Intelligence Learning Intention Scale [15] focuses on general university students. Although these instruments are valuable within their intended scope, none were designed specifically for clinical nurses, which may limit sensitivity to the unique demands of clinical practice.

These demands include high-frequency bedside decision-making, safety-critical documentation and handovers, and professional accountability in verifying clinical information. In contrast, the GenAILS addresses these gaps by incorporating dimensions such as Risk Identification, Critical Evaluation, and Ethics and Law. Considering the risks of GenAI hallucinations and bias, inclusion of risk identification is important for safeguarding patient safety, which is less explicit in general AI literacy tools [1,21,27]. The core contribution of this study lies in conceptualizing GenAI applications within specific clinical nursing contexts rather than remaining at a broad theoretical level, thereby enabling more precise identification of competency needs in real-world practice.

### Implications for Nursing Practice, Education, and Policy

The impact of GenAI on the nursing profession is multifaceted. In the educational domain, GenAI helps nursing learners resolve queries, understand complex concepts, and engage in problem formulation and clarification, thereby enhancing learning experiences and fostering critical thinking skills [12,61]. Without adequate GenAI literacy, learners risk applying erroneous content directly, which may lead to misjudgment, and may fail to detect misinformation or inherent biases in generated outputs [10,13]. In clinical practice, GenAI can be applied to documentation, creation of health education materials, design of individualized care plans, and development of realistic clinical simulation scenarios [11]. These applications can enhance workflow efficiency, creativity, and problem-solving effectiveness [1]. Failure to validate the accuracy of generated content poses significant risks to patient safety because of model hallucinations [7]. Overreliance on AI may also compromise interpersonal communication and erode clinical judgment [61].

The GenAILS items are grounded in real-world clinical task contexts, including documentation, decision support, and patient education, while emphasizing risk identification and professional accountability. From an implementation perspective, the GenAILS can support nursing managers in identifying training needs and prioritizing competency development, while assisting educators in designing evidence-based curricula and competency-based assessments. From a research perspective, the GenAILS offers a nurse-specific, standardized outcome measure for benchmarking GenAI literacy and evaluating educational interventions.

The total score provides an overall indicator of nurses’ self-perceived GenAI literacy, whereas subscale scores provide a competency profile across the six dimensions. Higher scores indicate higher levels of perceived GenAI literacy. For example, lower scores in Fundamental Knowledge may indicate the need for introductory GenAI education, whereas lower scores in Risk Identification, Critical Evaluation, or Ethics and Law may suggest the need for scenario-based training focused on hallucination detection, privacy protection, accountability, and safe clinical decision-making. GenAILS scores should not be used to classify nurses as having “adequate” or “inadequate” GenAI literacy. Rather, the scale is intended for benchmarking, educational needs assessment, curriculum planning, and intervention evaluation.

In program evaluation or implementation studies, the GenAILS can be used to compare cohorts, such as novice and experienced nurses, different clinical units, or different training formats.

Educational institutions should transition from traditional passive instruction to interactive, AI-integrated pedagogies [62,63]. The 6 dimensions of the GenAILS can be integrated into nursing curricula as core learning objectives. Teaching strategies should incorporate real-world clinical scenarios, such as identifying errors in AI-generated nursing documentation, to strengthen students’ decision-making capabilities in complex environments. Successful AI adoption relies heavily on educator support and appropriate pedagogical strategies [5,64,65].

At the curriculum and policy levels, its dimensions correspond to guidelines set by the Tertiary Education Quality and Standards Agency in Australia [21]. “Fundamental Knowledge,” “Responsible Use,” and “Critical Evaluation” align with foundational learning, support for applications in patient education, and requirements for clinical reflection on AI outputs, respectively. The findings align with international and local nursing profession development directions, supporting the GenAILS’s utility for curriculum development and outcome assessment.

Ultimately, the core value of nurses in the AI era lies in exercising human attributes, critical thinking, ethical judgment, and safeguarding patient safety rather than competing with machines in computational power. The GenAILS can serve as a common reference framework for clinical practice and academia, guiding the nursing profession to use GenAI safely, responsibly, and critically to enhance care quality in a rapidly evolving health care ecosystem.

Policymakers and regulatory bodies can use GenAI literacy competency frameworks to establish clear guidelines that promote responsible and ethical use. Policies that fail to keep pace with technological advancement may be ineffective in regulating GenAI’s rapid evolution and cross-disciplinary application and may exacerbate digital inequality and discrimination [1,10]. Equity considerations must also be addressed. Institutions should ensure equitable access to AI tools for faculty and students to mitigate the digital divide and prevent disparities in educational resource allocation. Investment in faculty development to remove technical barriers is essential for fairness and cultivating a workforce capable of responsible professional integration [18].

### Limitations and Future Directions

Despite the rigorous development process and demonstrated structural stability, several limitations should be acknowledged. The use of an online survey with convenience sampling and gift voucher incentives may have introduced self-selection bias and may have increased the possibility of attempted participation by ineligible individuals before data screening. Although data quality control procedures were implemented, nurses who voluntarily participated may have had greater interest in, familiarity with, or confidence in technology and AI. This bias may have led to higher self-reported GenAILS scores, potentially explaining the slightly negatively skewed item response distributions observed in this study.

The slightly negatively skewed item distributions may reflect genuine sample characteristics, such as higher baseline interest in or familiarity with GenAI among respondents. Although the extreme-group comparison indicated that the items retained discriminatory ability in this sample, this response pattern may also suggest a mild ceiling tendency, which could limit the scale’s ability to further differentiate nurses with moderate-to-high levels of self-perceived GenAI literacy. Therefore, at this stage, the GenAILS may be most appropriate for identifying core GenAI literacy competencies, assessing educational needs, and providing baseline measurements for clinical teaching and training, rather than serving as the sole tool for fine-grained differentiation among individuals with high GenAI literacy. Future studies should include nurses with varying levels of GenAI exposure or formal GenAI training to further examine the stability of the GenAILS factor structure and score distributions across diverse clinical settings and educational backgrounds. Additional research should also evaluate whether more challenging items or item response theory analyses are needed to improve discrimination at the upper end of the construct. In addition, because the GenAILS was developed and administered in Traditional Chinese within the Taiwanese nursing context, use of the GenAILS outside Taiwan requires rigorous translation and cross-cultural adaptation procedures.

Item No.36 (new item number 10) showed a relatively lower CFA standardized loading of 0.58, although its EFA loading was 0.66. Given its clinical importance for GenAI safety, the item was retained; however, together with the AVE of 0.45 for the Risk Identification dimension, its performance should be monitored and potentially refined in future validation studies. In addition, although the HTMT value between Risk Identification and Ethics and Law remained below the conservative threshold, its proximity to 0.85 suggests that future studies should examine whether these two dimensions remain distinct across diverse nursing populations and levels of GenAI exposure.

Another limitation concerns the self-report nature of the GenAILS. The scale assesses nurses’ self-perceived GenAI literacy rather than actual demonstrated competence or directly observed performance. Therefore, GenAILS scores should be interpreted as perceived literacy and may be influenced by response biases, including social desirability and overconfidence, particularly because GenAI literacy is an emerging and socially valued competency in health care. In addition, this study did not examine known-groups validity, such as comparisons between nurses with and without formal GenAI training or across different levels of clinical and GenAI-related experience. Future studies should further validate the GenAILS by incorporating known-groups comparisons, such as evaluating nurses’ ability to identify hallucinations, detect biased outputs, and appropriately verify GenAI-generated clinical content.

Temporal stability was not examined because test-retest data were not collected. Although the GenAILS demonstrated strong internal consistency and split-half reliability, these indices do not provide evidence of score stability over time. Future studies should evaluate test-retest reliability across an appropriate retest interval to determine whether the scale produces stable scores when nurses’ GenAI literacy is expected to remain unchanged.

Technological timeliness is an inherent challenge in GenAI research. Although the GenAILS is based on current technology and clinical scenarios, the applicability of certain items may diminish as new models and ethical issues emerge. Future research should adopt a rolling revision approach to update items in accordance with technological advancements and evolving clinical use cases.

### Conclusions

Drawing on evidence from the clinical, educational, and research domains, this study underscores the growing importance of GenAI literacy for the nursing profession. The GenAILS was developed with three key contributions. First, in terms of clinical applicability, the GenAILS is a practice-grounded instrument aligned with real-world clinical tasks to assess competencies relevant to patient safety and clinical decision-making in routine care. Second, in terms of educational relevance, it comprehensively covers responsible use, updated competencies, risk identification, fundamental knowledge, critical evaluation, and ethics and law, thereby mitigating the risk of “technical proficiency without professional judgment.” Third, in terms of research advancement, it provides standardized, quantifiable metrics that help bridge empirical gaps among educational outcomes, clinical implementation, and academic research.

## Appendix

Multimedia Appendix 1 Generative Artificial Intelligence Literacy Scale for Nurses-content validity.

Multimedia Appendix 2 Generative Artificial Intelligence Literacy Scale for Nurses-item analysis.

Multimedia Appendix 3 Generative Artificial Intelligence Literacy Scale for Nurses-GAILS scale.

## Abbreviations

AGFI: adjusted goodness-of-fit index
AI: artificial intelligence
AVE: average variance extracted
CFA: confirmatory factor analysis
CFI: comparative fit index
CHERRIES: Checklist for Reporting Results of Internet E-Surveys
COSMIN: Consensus-Based Standards for the Selection of Health Measurement Instruments
CR: composite reliability
EFA: exploratory factor analysis
GenAI: generative artificial intelligence
GenAILS: Generative Artificial Intelligence Literacy Scale for Nurses
GFI: goodness-of-fit index
HTMT: heterotrait-monotrait ratio
I-CVI: item-level content validity index
KMO: Kaiser-Meyer-Olkin
NNFI: nonnormed fit index
PAF: principal axis factoring
PNFI: parsimony normed fit index
RMSEA: root-mean-square error of approximation
SRMR: standardized root-mean-square residual

## Data Availability

The data used in this study are available from the corresponding author upon reasonable request.
