# Supplementary material for: Generative Artificial Intelligence Literacy Scale for Nurses: Development and Psychometric Evaluation
Source: J Med Internet Res. 2026 Jul 6;28:e95547. doi: 10.2196/95547 (PMC13386122; doi:10.2196/95547)
Supplement: Multimedia Appendix 1 [file jmir_v28i1e95547_app1.docx]

### **Multimedia Appendix 1**

### **Content validity: expert validity scores and experts’ suggested revision**

| No. | Item | Scoring criteria | Expert validity score | I-CVI | Experts’ suggested revisions |
| --- | --- | --- | --- | --- | --- |
| 1 | I understand the differences between GenAI and traditional search engines in how they produce information. | Relevance | 1 | 0.93 | I understand the differences between GenAI and traditional keyword-based search engines (e.g., web search) in how they generate information. |
|  |  | Clarity | 0.8 |  |  |
|  |  | Necessity | 1 |  |  |
| 2 | I understand the differences between GenAI and Discriminative AI in clinical applications. | Relevance | 0.8 | 0.8 | I understand the differences between GenAI and Discriminative AI in clinical applications. |
|  |  | Clarity | 0.8 |  |  |
|  |  | Necessity | 0.8 |  |  |
| 3 | I understand the functional differences among commonly used GenAI tools (e.g., ChatGPT, Gemini, Perplexity, Claude, Canva, Liner, and Gamma). | Relevance | 1 | 1 | I understand the functional differences among common GenAI tools. |
|  |  | Clarity | 1 |  |  |
|  |  | Necessity | 1 |  |  |
| 4 | I understand how GenAI is trained on large datasets and uses prompts to generate responses. | Relevance | 1 | 0.93 | I understand how GenAI undergoes model training using massive datasets.  I understand how GenAI generates responses through prompts. |
|  |  | Clarity | 0.8 |  |  |
|  |  | Necessity | 1 |  |  |
| 5 | I understand common uses of GenAI in clinical care (e.g., shift-handover summaries and patient education materials). | Relevance | 1 | 1 | *uncensored* |
|  |  | Clarity | 1 |  |  |
|  |  | Necessity | 1 |  |  |
| 6 | I understand that GenAI outputs may be influenced by the completeness and accuracy of the input information. | Relevance | 1 | 1 | *uncensored* |
|  |  | Clarity | 1 |  |  |
|  |  | Necessity | 1 |  |  |
| 7 | I understand that GenAI can convert patient data into structured information (e.g., health monitoring summaries). | Relevance | 1 | 0.93 | I understand that GenAI can organize a patient’s raw records (numeric data, text, and images) into individualized clinical information. |
|  |  | Clarity | 0.8 |  |  |
|  |  | Necessity | 1 |  |  |
| 8 | I can use GenAI to assist in writing nursing documentation. | Relevance | 1 | 1 | *uncensored* |
|  |  | Clarity | 1 |  |  |
|  |  | Necessity | 1 |  |  |
| 9 | I can use GenAI to help organize clinical information (e.g., shift-handover summaries and key points for clinical decision-making). | Relevance | 1 | 1 | I can use GenAI to assist in organizing clinical information (e.g., shift-handover summaries and key points for clinical decision-making). |
|  |  | Clarity | 1 |  |  |
|  |  | Necessity | 1 |  |  |
| 10 | I can use GenAI to create nursing education presentations and teaching materials. | Relevance | 1 | 1 | I can use GenAI to assist in creating nursing educational materials. |
|  |  | Clarity | 1 |  |  |
|  |  | Necessity | 1 |  |  |
| 11 | I can use GenAI to draft administrative reports or proposals (e.g., nursing quality improvement reports and patient safety incident reports). | Relevance | 1 | 1 | I can use GenAI to assist in generating administrative reports. |
|  |  | Clarity | 1 |  |  |
|  |  | Necessity | 1 |  |  |
| 12 | I can use GenAI to generate individualized patient education handouts based on a patient’s condition. | Relevance | 1 | 1 | I can use GenAI to design individualized health education content based on the patient's condition. |
|  |  | Clarity | 1 |  |  |
|  |  | Necessity | 1 |  |  |
| 13 | I can use GenAI to obtain specific suggestions for patient care as a reference. | Relevance | 1 | 0.93 | I can use GenAI to provide specific care recommendations as a clinical reference. |
|  |  | Clarity | 0.8 |  |  |
|  |  | Necessity | 1 |  |  |
| 14 | I can use GenAI to facilitate collaboration and communication within the interprofessional healthcare team. | Relevance | 1 | 1 | Based on expert feedback, this content was merged into Item 9. |
|  |  | Clarity | 1 |  |  |
|  |  | Necessity | 1 |  |  |
| 15 | I can use GenAI-generated clinical data visualizations to support the development of a patient’s nursing care plan. | Relevance | 1 | 1 | I can use GenAI to generate clinical data charts to assist in formulating patient care plans. |
|  |  | Clarity | 1 |  |  |
|  |  | Necessity | 1 |  |  |
| 16 | I can select appropriate GenAI tools for different tasks. | Relevance | 0.8 | 0.87 | I can provide clear and specific instructions when using GenAI to improve the accuracy and usefulness of its outputs. |
|  |  | Clarity | 0.8 |  |  |
|  |  | Necessity | 1 |  |  |
| 17 | I can provide clear and specific prompts when using GenAI to improve the accuracy and usefulness of its outputs. | Relevance | 0.6 | 0.53 | The item was deleted because of a low I-CVI and expert feedback regarding unclear semantics and redundancy with similar items. |
|  |  | Clarity | 0.6 |  |  |
|  |  | Necessity | 0.4 |  |  |
| 18 | I can critically evaluate the clinical recommendations provided by GenAI. | Relevance | 1 | 0.93 | I can critically evaluate whether GenAI-generated clinical recommendations are clinically sound. |
|  |  | Clarity | 0.8 |  |  |
|  |  | Necessity | 1 |  |  |
| 19 | I can critically evaluate whether GenAI-generated content aligns with a patient’s individual needs. | Relevance | 1 | 0.93 | I can critically evaluate whether the nursing guidance produced by GenAI meets individual patient needs. |
|  |  | Clarity | 0.8 |  |  |
|  |  | Necessity | 1 |  |  |
| 20 | I can critically evaluate whether GenAI-generated nursing documentation is clinically applicable. | Relevance | 1 | 0.87 | I can critically evaluate the clinical applicability of nursing documentation generated by GenAI. |
|  |  | Clarity | 0.6 |  |  |
|  |  | Necessity | 1 |  |  |
| 21 | I can critically compare GenAI-generated information with clinical evidence and make a judgment. | Relevance | 1 | 0.93 | I can make critical decisions when GenAI outputs conflict with clinical professional judgment. |
|  |  | Clarity | 0.8 |  |  |
|  |  | Necessity | 1 |  |  |
| 22 | When GenAI outputs are inconsistent with my clinical judgment, I can critically evaluate whether the generated content is clinically sound. | Relevance | 1 | 0.93 | I can determine whether to modify or discard GenAI recommendations based on the clinical context. |
|  |  | Clarity | 0.8 |  |  |
|  |  | Necessity | 1 |  |  |
| 23 | I can critically evaluate GenAI outputs and propose revisions. | Relevance | 0.8 | 0.8 | I can critically evaluate GenAI recommendations and, based on the clinical context, decide whether they should be revised or disregarded. |
|  |  | Clarity | 0.8 |  |  |
|  |  | Necessity | 0.8 |  |  |
| 24 | I can critically evaluate GenAI outputs and preliminarily identify possible causes of errors or misinformation. | Relevance | 0.8 | 0.8 | I can critically evaluate the causes of incorrect information generated by GenAI (e.g., input errors, data bias, or model limitations). |
|  |  | Clarity | 0.8 |  |  |
|  |  | Necessity | 0.8 |  |  |
| 25 | I can critically evaluate GenAI outputs and, based on professional judgment, revise them or decide not to use them. | Relevance | 0.8 | 0.8 | Based on expert feedback, the revised content was merged into Item 22. |
|  |  | Clarity | 0.8 |  |  |
|  |  | Necessity | 0.8 |  |  |
| 26 | When GenAI-generated recommendations do not match real-world clinical situations, I can identify key missing information in the generated content. | Relevance | 1 | 1 | *uncensored* |
|  |  | Clarity | 1 |  |  |
|  |  | Necessity | 1 |  |  |
| 27 | When GenAI-generated recommendations do not match real-world clinical situations, I can proactively provide necessary clinical information to revise the recommendations. | Relevance | 1 | 1 | When GenAI-generated recommendations do not match real-world clinical situations, I can proactively provide necessary clinical information. |
|  |  | Clarity | 1 |  |  |
|  |  | Necessity | 1 |  |  |
| 28 | When GenAI-generated recommendations do not match real-world clinical situations, I use other reliable sources to cross-check the information. | Relevance | 1 | 1 | When GenAI-generated recommendations do not match real-world clinical situations, I use credible sources to cross-check the reliability of the generated content. |
|  |  | Clarity | 1 |  |  |
|  |  | Necessity | 1 |  |  |
| 29 | When GenAI-generated recommendations do not match real-world clinical situations, I can use my clinical experience to make appropriate judgments and adjustments. | Relevance | 1 | 0.8 | When GenAI-generated recommendations do not match real-world clinical situations, I can use my clinical experience to assess the content and make necessary adjustments. |
|  |  | Clarity | 0.8 |  |  |
|  |  | Necessity | 0.6 |  |  |
| 30 | When there are concerns about data leakage or information security related to GenAI, I know how to take appropriate data protection measures. | Relevance | 1 | 1 | When there are concerns about data leakage or information security related to GenAI, I know how to take appropriate data protection measures. |
|  |  | Clarity | 1 |  |  |
|  |  | Necessity | 1 |  |  |
| 31 | When there are concerns about data leakage or information security related to GenAI, I understand the relevant legal responsibilities. | Relevance | 1 | 1 | When there are concerns about data leakage or information security related to GenAI, I understand the relevant legal responsibilities. |
|  |  | Clarity | 1 |  |  |
|  |  | Necessity | 1 |  |  |
| 32 | When there are concerns about data leakage or information security related to GenAI, I recognize that patient privacy may be at risk. | Relevance | 1 | 1 | When there are concerns about data leakage or information security related to GenAI, I can identify potential risks to patient privacy. |
|  |  | Clarity | 1 |  |  |
|  |  | Necessity | 1 |  |  |
| 33 | When GenAI is incorporated into care-related decision-making, I can clearly explain the patient’s autonomy. | Relevance | 0.8 | 0.73 | When GenAI is incorporated into care-related decision-making, I can clearly explain that the patient has the right to accept or refuse the use of AI-assisted care. |
|  |  | Clarity | 0.6 |  |  |
|  |  | Necessity | 0.8 |  |  |
| 34 | When GenAI is incorporated into care-related decision-making, I can clearly explain GenAI’s supportive role and its limitations. | Relevance | 1 | 1 | When GenAI is incorporated into care-related decision-making, I can clearly explain the support GenAI provides and its limitations. |
|  |  | Clarity | 1 |  |  |
|  |  | Necessity | 1 |  |  |
| 35 | When GenAI is incorporated into care-related decision-making, I can comply with legal requirements for AI-related data processing in healthcare settings (e.g., patient data protection laws). | Relevance | 1 | 1 | When GenAI is involved in care decisions, I comply with legal regulations regarding data processing in clinical settings. |
|  |  | Clarity | 1 |  |  |
|  |  | Necessity | 1 |  |  |
| 36 | When GenAI is incorporated into care-related decision-making, I understand that I remain professionally accountable and may face legal risks if it contributes to an incorrect decision. | Relevance | 1 | 0.93 | When GenAI is involved in care decisions, I understand that healthcare professionals bear the ultimate decision-making responsibility and legal liability. |
|  |  | Clarity | 0.8 |  |  |
|  |  | Necessity | 1 |  |  |
| 37 | When GenAI is incorporated into care-related decision-making, I can identify potential ethical issues in the generated content. | Relevance | 0.8 | 0.8 | When GenAI is involved in care decisions, I can identify potential ethical controversies in the generated content |
|  |  | Clarity | 0.8 |  |  |
|  |  | Necessity | 0.8 |  |  |
| 38 | I can identify errors, hallucinations, and logical issues in GenAI-generated content. | Relevance | 1 | 0.93 | I can identify hallucinations within the data generated by GenAI. |
|  |  | Clarity | 0.8 |  |  |
|  |  | Necessity | 1 |  |  |
| 39 | I can identify potential patient safety risks in recommendations generated by GenAI. | Relevance | 1 | 1 | I can identify potential patient safety risks in care recommendations generated by GenAI. |
|  |  | Clarity | 1 |  |  |
|  |  | Necessity | 1 |  |  |
| 40 | I can assess the interpretability and transparency of GenAI-generated information. | Relevance | 1 | 0.87 | I can identify whether the data generated by GenAI possesses clear and verifiable sources. |
|  |  | Clarity | 0.6 |  |  |
|  |  | Necessity | 1 |  |  |
| 41 | I can identify risks resulting from erroneous reasoning by GenAI | Relevance | 1 | 1 | I can identify risks resulting from erroneous reasoning by GenAI. |
|  |  | Clarity | 1 |  |  |
|  |  | Necessity | 1 |  |  |
| 42 | I can identify potential bias or underrepresentation in the training data used for GenAI. | Relevance | 1 | 1 | I can identify potential errors or underrepresentation in the training data used for GenAI. |
|  |  | Clarity | 1 |  |  |
|  |  | Necessity | 1 |  |  |
| 43 | I can assess GenAI’s applicability and potential bias across different populations (e.g., by age or health condition). | Relevance | 1 | 0.93 | I can identify the applicability and potential biases of GenAI across different population groups. |
|  |  | Clarity | 0.8 |  |  |
|  |  | Necessity | 1 |  |  |
| 44 | I proactively learn to use GenAI to improve clinical nursing care quality. | Relevance | 0.8 | 0.8 | I actively learn practical applications of GenAI to enhance the quality of clinical care. |
|  |  | Clarity | 0.8 |  |  |
|  |  | Necessity | 0.8 |  |  |
| 45 | I actively keep up with the rapid changes in the clinical application of GenAI | Relevance | 0.8 | 0.8 | I actively keep up with the rapid changes in the clinical application of GenAI |
|  |  | Clarity | 0.8 |  |  |
|  |  | Necessity | 0.8 |  |  |
| 46 | I proactively participate in relevant learning activities (e.g., workshops, tutorial videos, or online resources) to enhance my understanding of GenAI. | Relevance | 1 | 0.6 | I continuously update my competencies in applying GenAI. |
|  |  | Clarity | 1 |  |  |
|  |  | Necessity | 1 |  |  |
| 47 | I proactively explore innovative ways to apply GenAI in clinical care. | Relevance | 0.8 | 0.8 | Based on expert feedback, the revised content was merged into Item 50. |
|  |  | Clarity | 0.8 |  |  |
|  |  | Necessity | 0.8 |  |  |
| 48 | When I encounter difficulties using GenAI, I proactively seek assistance. | Relevance | 1 | 0.87 | I proactively seek assistance when encountering difficulties in using GenAI. |
|  |  | Clarity | 0.6 |  |  |
|  |  | Necessity | 1 |  |  |
| 49 | I regularly review my use of GenAI and address areas for improvement. | Relevance | 0.8 | 0.8 | Based on expert feedback, the revised content was merged into Item 46. |
|  |  | Clarity | 0.6 |  |  |
|  |  | Necessity | 1 |  |  |
| 50 | When GenAI introduces new features or updates, I learn how to use them. | Relevance | 0.8 | 0.87 | I actively learn new applications and innovative methods of GenAI. |
|  |  | Clarity | 0.8 |  |  |
|  |  | Necessity | 1 |  |  |
| S-CVI/Ave：0.917 | | | | | |
