# Supplementary material for: Generative Artificial Intelligence Literacy Scale for Nurses: Development and Psychometric Evaluation
Source: J Med Internet Res. 2026 Jul 6;28:e95547. doi: 10.2196/95547 (PMC13386122; doi:10.2196/95547)
Supplement: Multimedia Appendix 2 [file jmir_v28i1e95547_app2.docx]

### **Multimedia Appendix 2**

### **Item analysis results**

| Item | Mean | Variance | Skewness | Kurtosis | Independent-samples t test (top/bottom 27%) | Corrected item–total correlation | Cronbach’s α if item deleted |
| --- | --- | --- | --- | --- | --- | --- | --- |
| 1 | 3.93 | 0.598 | -0.640 | 0.863 | <.001 | 0.469 | 0.955 |
| 2 | 3.76 | 0.775 | -0.489 | -0.020 | <.001 | 0.561 | 0.955 |
| 3 | 3.75 | 0.787 | -0.341 | -0.368 | <.001 | 0.563 | 0.955 |
| 4 | 3.93 | 0.678 | -0.558 | 0.118 | <.001 | 0.486 | 0.955 |
| 5 | 3.86 | 0.711 | -0.577 | 0.249 | <.001 | 0.514 | 0.955 |
| 6 | 3.88 | 0.677 | -0.440 | -0.203 | <.001 | 0.549 | 0.955 |
| 7 | 4.10 | 0.559 | -0.514 | -0.008 | <.001 | 0.426 | 0.955 |
| 8 | 4.01 | 0.545 | -0.498 | 0.172 | <.001 | 0.468 | 0.955 |
| 9 | 3.66 | 0.781 | -0.561 | 0.343 | <.001 | 0.523 | 0.955 |
| 10 | 3.79 | 0.793 | -0.615 | 0.300 | <.001 | 0.577 | 0.955 |
| 11 | 3.94 | 0.665 | -0.579 | 0.391 | <.001 | 0.572 | 0.955 |
| 12 | 3.87 | 0.749 | -0.522 | 0.054 | <.001 | 0.584 | 0.955 |
| 13 | 3.81 | 0.747 | -0.428 | -0.129 | <.001 | 0.607 | 0.955 |
| 14 | 3.85 | 0.671 | -0.402 | -0.089 | <.001 | 0.611 | 0.955 |
| 15 | 3.86 | 0.700 | -0.543 | 0.185 | <.001 | 0.639 | 0.954 |
| 16 | 3.84 | 0.696 | -0.461 | 0.074 | <.001 | 0.623 | 0.954 |
| 17 | 3.85 | 0.578 | -0.285 | -0.159 | <.001 | 0.547 | 0.955 |
| 18 | 3.93 | 0.515 | -0.376 | 0.260 | <.001 | 0.568 | 0.955 |
| 19 | 3.92 | 0.552 | -0.309 | -0.049 | <.001 | 0.579 | 0.955 |
| 20 | 3.79 | 0.615 | -0.156 | -0.355 | <.001 | 0.606 | 0.955 |
| 21 | 3.89 | 0.578 | -0.283 | -0.147 | <.001 | 0.634 | 0.954 |
| 22 | 3.89 | 0.673 | -0.447 | -0.004 | <.001 | 0.586 | 0.955 |
| 23 | 3.91 | 0.585 | -0.385 | 0.134 | <.001 | 0.609 | 0.955 |
| 24 | 3.88 | 0.578 | -0.324 | -0.063 | <.001 | 0.566 | 0.955 |
| 25 | 3.96 | 0.509 | -0.312 | 0.010 | <.001 | 0.572 | 0.955 |
| 26 | 3.90 | 0.509 | -0.329 | 0.042 | <.001 | 0.491 | 0.955 |
| 27 | 4.02 | 0.472 | -0.308 | -0.025 | <.001 | 0.559 | 0.955 |
| 28 | 3.60 | 0.927 | -0.440 | -0.206 | <.001 | 0.508 | 0.955 |
| 29 | 3.61 | 1.028 | -0.413 | -0.452 | <.001 | 0.540 | 0.955 |
| 30 | 3.93 | 0.654 | -0.512 | 0.261 | <.001 | 0.575 | 0.955 |
| 31 | 3.93 | 0.568 | -0.283 | -0.247 | <.001 | 0.526 | 0.955 |
| 32 | 3.91 | 0.593 | -0.485 | 0.312 | <.001 | 0.596 | 0.955 |
| 33 | 3.99 | 0.524 | -0.294 | -0.170 | <.001 | 0.549 | 0.955 |
| 34 | 3.94 | 0.625 | -0.523 | 0.287 | <.001 | 0.560 | 0.955 |
| 35 | 3.90 | 0.578 | -0.299 | -0.142 | <.001 | 0.571 | 0.955 |
| 36 | 3.84 | 0.562 | -0.300 | -0.006 | <.001 | 0.528 | 0.955 |
| 37 | 3.91 | 0.522 | -0.310 | 0.018 | <.001 | 0.568 | 0.955 |
| 38 | 3.80 | 0.659 | -0.359 | 0.012 | <.001 | 0.582 | 0.955 |
| 39 | 3.89 | 0.536 | -0.381 | 0.219 | <.001 | 0.544 | 0.955 |
| 40 | 3.88 | 0.593 | -0.374 | 0.106 | <.001 | 0.575 | 0.955 |
| 41 | 3.86 | 0.615 | -0.469 | 0.334 | <.001 | 0.557 | 0.955 |
| 42 | 3.97 | 0.532 | -0.441 | 0.370 | <.001 | 0.497 | 0.955 |
| 43 | 4.00 | 0.562 | -0.375 | -0.088 | <.001 | 0.534 | 0.955 |
| 44 | 4.01 | 0.541 | -0.309 | -0.257 | <.001 | 0.565 | 0.955 |
| 45 | 4.00 | 0.451 | -0.234 | -0.089 | <.001 | 0.532 | 0.955 |
| 46 | 4.01 | 0.500 | -0.236 | -0.346 | <.001 | 0.503 | 0.955 |
