# Supplementary material for: Generative Artificial Intelligence Literacy Scale for Nurses: Development and Psychometric Evaluation
Source: J Med Internet Res. 2026 Jul 6;28:e95547. doi: 10.2196/95547 (PMC13386122; doi:10.2196/95547)
Supplement: Multimedia Appendix 3 [file jmir_v28i1e95547_app3.docx]

**Multimedia Appendix 3**

**The Generative Artificial Intelligence Literacy Scale for Nurses**

| Factor | Item No | New  Item | Item Description |
| --- | --- | --- | --- |
| Responsible Use | 11 | 1 | I can use GenAI to assist in creating nursing educational materials |
|  | 12 | 2 | I can use GenAI to assist in generating administrative reports |
|  | 13 | 3 | I can use GenAI to design individualized health education content based on the patient’s condition |
|  | 14 | 4 | I can use GenAI to provide specific care recommendations as a clinical reference |
|  | 15 | 5 | I can use GenAI to generate clinical data charts to assist in formulating patient care plans |
| Updated Competencies | 43 | 6 | I actively keep up with rapid changes in the clinical application of GenAI |
|  | 44 | 7 | I continuously update my competencies in applying GenAI |
|  | 45 | 8 | I proactively seek assistance when encountering difficulties in using GenAI |
|  | 46 | 9 | I actively learn new applications and innovative methods of GenAI |
| Risk Identification | 36 | 10 | I can identify hallucinations in data generated by GenAI |
|  | 39 | 11 | I can identify risks resulting from erroneous reasoning by GenAI |
|  | 40 | 12 | I can identify potential inaccuracies or lack of representativeness in the training data used by GenAI. |
|  | 41 | 13 | I can identify the applicability and potential biases of GenAI across different population groups |
| Fundamental knowledge | 2 | 14 | I understand the differences between GenAI and discriminative AI in clinical applications |
|  | 3 | 15 | I understand the functional differences among common GenAI tools |
|  | 4 | 16 | I understand how GenAI undergoes model training using massive datasets |
|  | 5 | 17 | I understand how GenAI generates responses through prompts |
| Critical Evaluation | 18 | 18 | I can critically evaluate whether the nursing guidance produced by GenAI meets the needs of individual patients |
|  | 19 | 19 | I can critically evaluate the clinical applicability of nursing documentation generated by GenAI |
|  | 21 | 20 | I can make critical decisions when GenAI outputs conflict with clinical professional judgment |
|  | 23 | 21 | I can determine whether to modify or discard GenAI recommendations based on the clinical context |
| Ethics & Law | 33 | 22 | When GenAI is involved in care decisions, I comply with legal regulations regarding data processing in clinical settings |
|  | 34 | 23 | When GenAI is involved in care decisions, I understand that healthcare professionals bear ultimate decision-making responsibility and legal liability |
|  | 35 | 24 | When GenAI is involved in care decisions, I can identify potential ethical controversies in generated content |
